# Enhancing the Surface Quality of Micro Titanium Alloy Specimen in WEDM Process by Adopting TGRA-Based Optimization

**DOI:** 10.3390/ma13061440

**Published:** 2020-03-21

**Authors:** Muthuramalingam Thangaraj, Ramamurthy Annamalai, Khaja Moiduddin, Mohammed Alkindi, Sundar Ramalingam, Osama Alghamdi

**Affiliations:** 1 Department of Mechatronics Engineering, SRM Institute of Science and Technology, Kattankulathur 603203, India; muthurat@srmist.edu.in; 2 Department of Mechanical Engineering, Saveetha Engineering College, Thandalam, Chennai 602105, India; ramapec@gmail.com; 3 Advanced Manufacturing Institute, King Saud University, Riyadh 11421, Saudi Arabia; 4 Department of Oral and Maxillofacial Surgery, College of Dentistry, King Saud University, Riyadh 11545, Saudi Arabia; malkindi@ksu.edu.sa (M.A.); smunusamy@ksu.edu.sa (S.R.); oghamdi@ksu.edu.sa (O.A.)

**Keywords:** EDM, surface, optimization, machining, titanium

## Abstract

The surface measures of machined titanium alloys as dental materials can be enhanced by adopting a decision-making algorithm in the machining process. The surface quality is normally characterized by more than one quality parameter. Hence, it is very important to establish multi-criteria decision making to compute the optimal process factors. In the present study, Taguchi–Grey analysis-based criteria decision making has been applied to the input process factors in the wire EDM (electric discharge machining) process. The recast layer thickness, wire wear ratio and micro hardness have been chosen to evaluate the quality measures. It was found that the wire electrode selection was the most influential factor on the quality measures in the WEDM process, due to its significance in creating spark energy. The optimal arrangement of the input process parameters has been found using the proposed approach as gap voltage (70 V), discharge current (15 A) and duty factor (0.6). It was proved that the proposed method can enhance the efficacy of the process. Utilizing the computed combination of optimal process parameters in surface quality analysis has significantly contributed to improving the quality of machining surface.

## 1. Introduction

Due to its unique physical properties such as higher corrosion resistance and considerable strength, titanium (α-β) alloy (Ti-6Al-4V) is employed in synthesizing dental specimens [1]. As a dental implant material, titanium alloy must possess an adequate surface quality, free from residual stress. It is very difficult to remove the material using traditional machining processes due its high strength, and as such, nontraditional material removal processes such as laser beam machining (LBM), hybrid machining, electro chemical machining (ECM), wire electrical discharge machining (WEDM) and abrasive-water jet machining (AWJM) are utilized. Titanium alloy as dental material should have an optimal surface finish through the machining process. The conventional machining method produces higher residual stress due to vibrations made during the process [2]. The LBM and hybrid machining processes produce a high heat affected zone (HZ) on the machined specimens [3]. The improper selection of laser power results in affecting the machining performance of titanium alloy in the LBM process [4]. The AWJM process causes the titanium alloy specimens to considerably taper [5]. The ECM process may result in the corrosion of the workpiece specimen [6]. For the utilization of titanium alloy as a bio material, the specimen should have an optimal surface finish and performance during the machining process [7]. The quality measures of the machined specimens should be as high as possible in order to manufacture the product with favorable performance measures. The WEDM process is widely used to machine titanium species as it produces relatively lower taperness and kerf widths. The material removal is achieved in this process by applying a pulsed DC supply between the workpiece and wire electrode in an insulated environment. As the WEDM process is of a nonlinear nature, the enhancement of process parameters is required to obtain better performance measures. The surface quality can be effectively controlled by white layer formation in the EDM process [8]. The surface quality performance measures are mostly influenced by enhancing the input process factors in WEDM. The optimization of input process factors in machining methods such as the WEDM process is very tedious due to their unsystematic nature [9]. It is important to establish multi-response optimization techniques to determine the optimal parameter combination in the WEDM process [10,11]. Many multiple performance decision-making techniques such as the assignment of the weight method, genetic algorithms, the Taguchi data envelopment analysis ranking (DEAR) method and the Taguchi–Grey relation analysis (TGRA) that are available can convert multiple response characteristics into a single performance measure in any process. Amongst these, TGRA is widely used as it has higher efficacy and easy adaptability. Nanthakumar et al. made an attempt to introduce the TGRA method as a means of optimizing process parameters in the materials development process. It has been found that the proposed method can significantly improve quality measures [12]. The optimal set of sintering process factors in the grinding process was found using the TGRA method. It has been observed that the TGRA method can determine the optimal combination effectively in any manufacturing process [13]. Pillai et al. effectively applied the TGRA method to optimize the parameters involved in the robotics-assisted machining process [14]. It was inferred that the TGRA method can compute the optimal process parameters, the significance of which determines responses in machining processes [15,16,17]. The grinding parameters of green manufacturing processes can be optimized using the TGRA method. It has been found that the proposed approach can increase prediction accuracy [18,19]. Product design can be further enhanced by the TGRA method [20]. The detailed survey showed that only multi-criteria decision making (MCDM) can provide better process factors in machining processes. It was also found that little attention was given to optimizing surface quality performance measures such as white layer thickness, wire wear ratio and micro hardness in the WEDM process of machining titanium alloy. In regards to structure, the surface should be of the highest possible quality. MCDM can be utilized in achieving this. In the present study, Taguchi’s experiment model and Grey’s relational analysis methodology were applied in order to enhance the surface performance measures in cutting titanium alpha-beta (Ti-6Al-4V) alloy with the WEDM process. The following are the primary aims of the investigation on machinability using various process factors:

1. To compute the optimal process factors for obtaining better surface quality measures of titanium alloy specimens using the TGRA method.
2. To evaluate the influence of input factors on surface measures.
3. To investigate the surface quality at optimal levels in the process.

## 2. Materials and Methods

Titanium(α-β) alloy was chosen as the specimen due to its usability as a dental implant material. Despite possessing a higher corrosion resistance and lighter weight, it is a high strength material [1]. The measurement approaches of quality measures and design of experiments are also discussed in the present subsection. Due to their efficacy in evaluating surface related parameters, pulse-on time (T_on_), Pulse-off time (T_off_), servo voltage (SV), wire electrode (WE) and wire tension (WT) were selected as the input factors of the multi criteria optimization in the present study. The selection of process factors is given in Table 1 [17].

The surface quality of machined workpiece specimens, average white layer thickness (AWLT), micro hardness (MH) and wire wear ratio (WWR) were selected as the surface measures in the present study. In the WEDM process, the machining quality of the specimen is considerably characterized by the wire wear ratio due to its importance in evaluating the discharge energy of every pulse cycle. WWR can be calculated using the following Equation (1): [20,21]

(1) $$\mathrm{WWR}=\frac{W_{I}-W_{F}}{W_{I}}$$

where W_I_-Initial weight of the workpiece specimen; W_F_-Final weight of the workpiece specimen after the machining process.

The weight of workpiece specimens was calculated using electronics balances with an accuracy of 0.001 g [22]. The micro hardness (HV) of the processed workpiece was computed using Vickers-based micro hardness tester in Kg/mm^2^. The applied load was considered as 300 g. Due to the divergent width of the AWLT over the machined surface, it must be taken for the purpose of analysis and was calculated using the Equation (2) as follows:

(2) $$\mathrm{AWLT}=\frac{\mathrm{Area}\;\mathrm{of}\;\mathrm{recast}\;\mathrm{layer}}{\mathrm{Length}\;\mathrm{of}\;\mathrm{recast}\;\mathrm{layer}}$$

**Figure 1:**
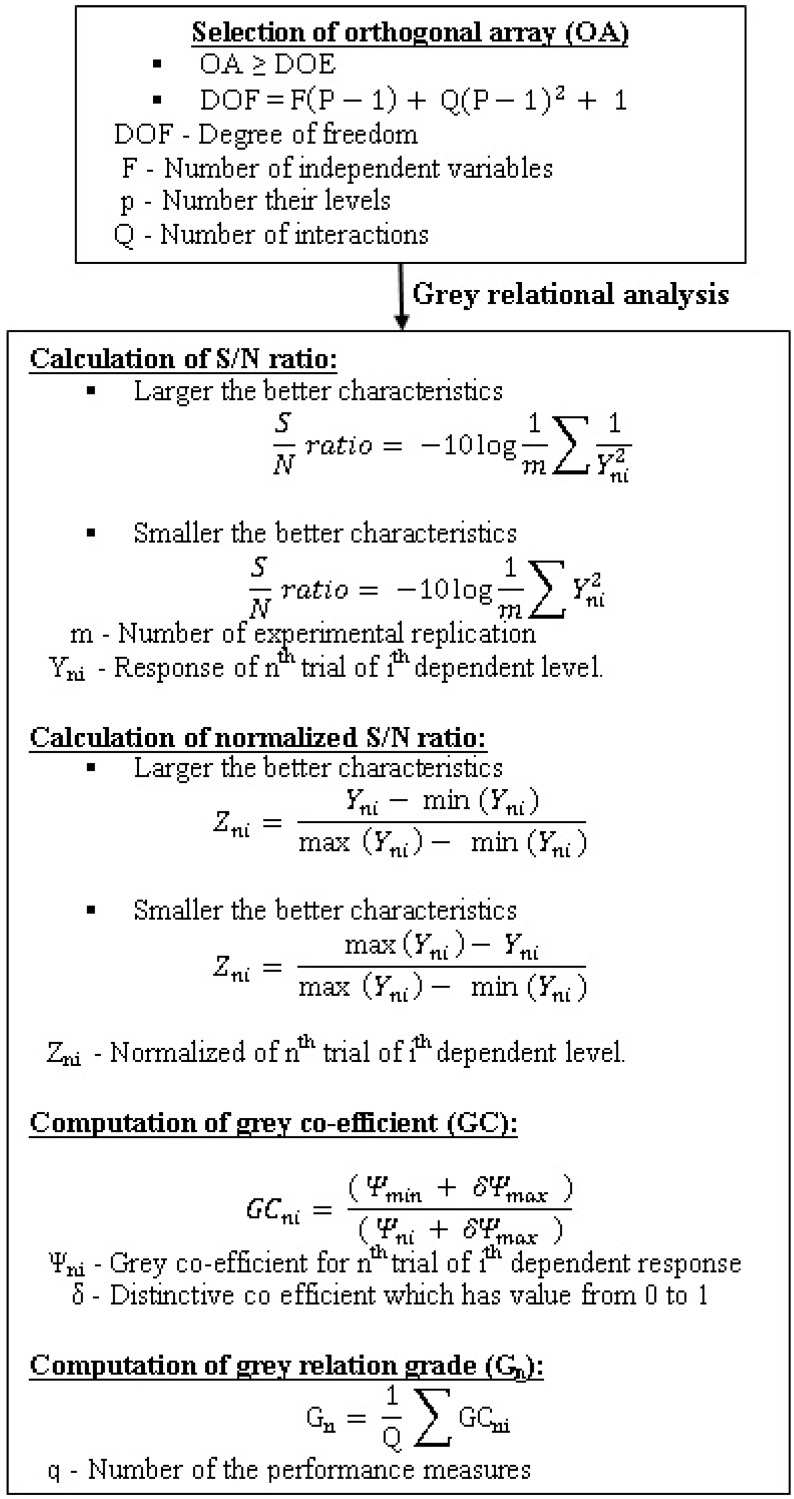
Steps involved in TGRA computation.

The AWLT area was computed by sketching a polyline along the white layer on the specimen using WEDM [8,23]. Figure 1 illustrates the steps involved in TGRA of the present work.

## 3. Results and Discussion

**Figure 2:**
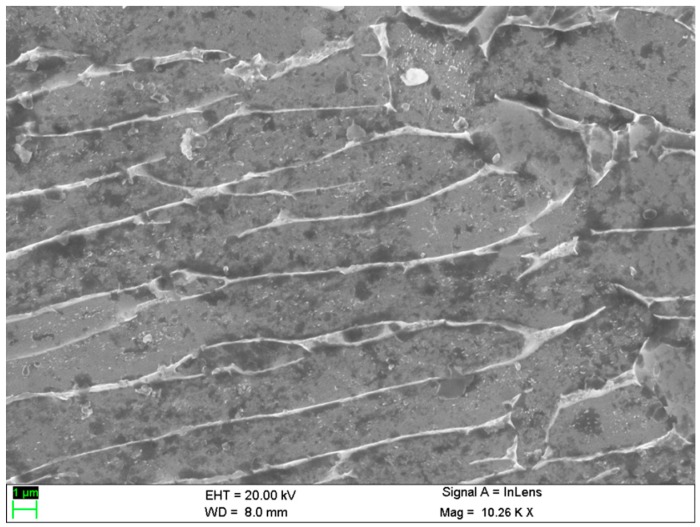
Surface topography of the machined Ti-6Al-4V alloy in WEDM process.

Titanium alloy specimens were machined using the WEDM method into rectangular specimens in accordance with the Taguchi system. The performance measures of each trial have been measured and tabulated. Figure 2 demonstrates the surface topography of a machined titanium alloy specimen in WEDM. In the EDM process, the surface morphology replicates the tool electrode. The surface patterns caused by the wire electrodes can be clearly viewed in the machined surface as shown in Figure 2. Table 2 shows the L_27_ orthogonal table with input factors and response values in the EDM process. Table 3 illustrates the signal-to-noise (S/N) ratio with their normalized value (N S/N) of the selected performance measures. Micro hardness was chosen as a larger-the-better (LTB) quality, whereas WWR and AWLT were chosen as smaller-the-better (STB) quality level characteristics. As the present study of surface performance measures was completed with both the LTB and STM quality characteristics, the distinguishing coefficient value was selected as 0.5 [6].

### 3.1. Computation of Optimal Process Parameters

The values of Grey Relational (GR) components along with their rank of all trials are given in Table 4. Table 5 shows the average of the GR scale for all the levels of process factors. The average Grey technique value specifies the relationship levels among the comparative values and a reference value. Hence, the optimal assessment of each process factor is the highest average GR value in the process. 

**Figure 3:**
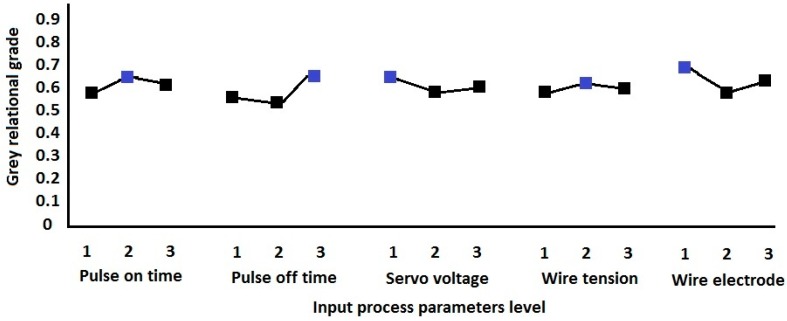
Based optimal process parameters computation.

Figure 3 shows the response graph of average Grey Relational grades. It was observed that the optimal values of parameters are level 2 (T_on_), level 3 (T_off_), level 1 (SV), level 2 (WT) and level 1 (WE). The high-low indicates the level of the most dominant process parameter in formulating the performance measures among all the input process parameters in any machining process. It was observed that the wire electrode significantly influences the quality measures such as white layer thickness, micro hardness and WWR in the WEDM process. The crater size produced by the discharge energy is mainly characterized by the electric current conductance of the electrode in WEDM. As the surface quality of the machined workpiece is evaluated using crater size and material removal, the wire electrode possesses a vital role in evaluating the surface performance measures in WEDM [22].

### 3.2. Confirmation Experiment

Following the detection of the optimal factor combination, the confirmation test was performed to examine its confidence. In this present test, the experiment was conducted in WEDM under the optimal factor combination [7]. The predicted GR grade (G_a_) was computed as per the following Equation (3):

(3) $$G_{a}=G_{b}+\sum \left(G_{c}-G_{b}\right)$$

where G_b_-total average GR grade and G_c_-optimal average GR grade. The predicted value was found as 0.762953. The response values were obtained with an optimal factor combination of 0.1675 (WWR), 512.8 (MH) and 4.35 (AWLT). The GR grade was calculated as 0.787367. The GR grade value was improved by 3.2% from the predicted mean value. 

**Figure 4:**
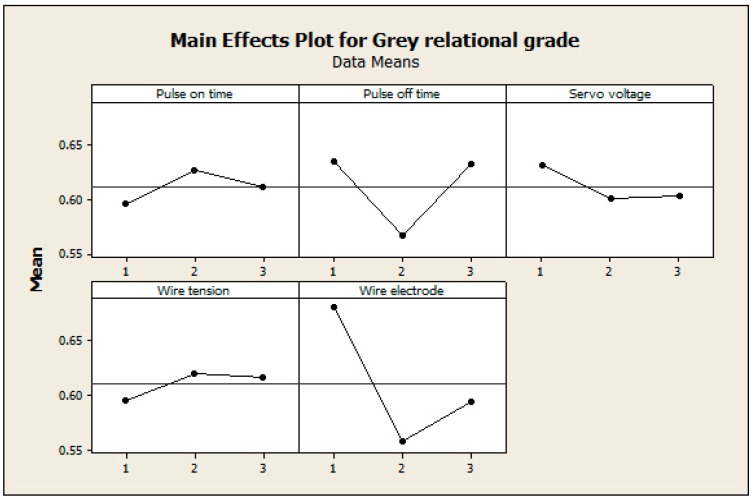
Effects of input process factors on Grey relational grade.

The main effect plot was used to examine the significance of the input factors on responses using Minitab software [16]. Figure 4 shows the effects of input factors on Grey relational grades. The surface measures of the machined specimens were considerably characterized by the wire wear ratio due to its significance in examining the discharge energy of every pulse cycle. The micro hardness and white layer thickness are characterized by the amount of resolidification of the workpiece and tool electrode. As the servo voltage contributes mostly to resolidification, it considerably influences the micro hardness and AWLT. The physical characteristics of the wire electrode influence the white layer thickness, as the recast layer consists of melted wire electrode material. The selection of the wire electrode has a vital role in determining the AWLT due to its weight in formulating the recast layer thickness. As the wire electrode has a considerable effect on determining the surface quality related parameters, it has a highly influential role in deciding surface measures in WEDM.

## 4. Surface Analysis under Optimal Process Parameters Combination

The surface quality measures of the processed specimen under the optimal input factors such as T_on_ (120 µs), T_off_ (50 µs), SV (40 V), WT (7 Kg) and WE (BWE) have been analyzed for surface morphology enhancement. 

**Figure 5:**
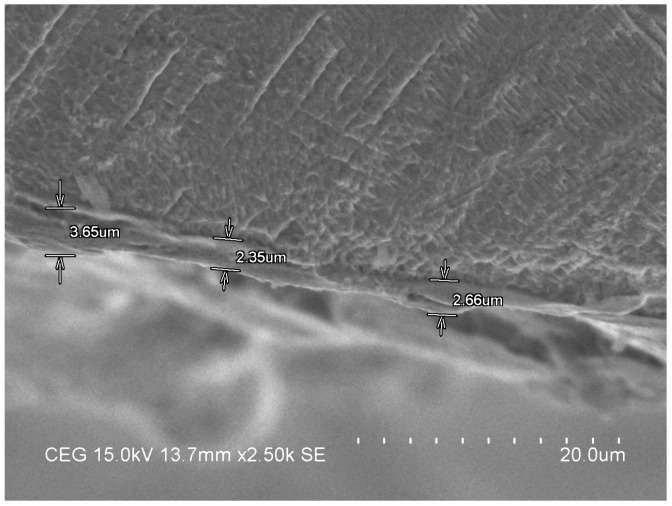
White layer distribution of machined specimen in WEDM process.

As surface hardness is the surface layer property, the AWLT has a highly influential role in determining the MH of the specimen. The recast layer of the processed specimen in the WEDM process should have a uniform and minimal thickness in order to enhance the quality measures. While the white layer formation cannot be avoided, its thickness should be as minimal as possible. The specimen processed under the optimal input parameters combination displays a uniform and low AWLT as shown in Figure 5. The electrical pulse energy is proportional to the width of the white layer. The pulse duration can increase the pulse energy in the WEDM process. The pulse energy impacts the resolidification of the melted particles. The pulse energy level as displayed in Figure 5 results in a lower recast layer thickness and uniform distribution.

**Figure 6:**
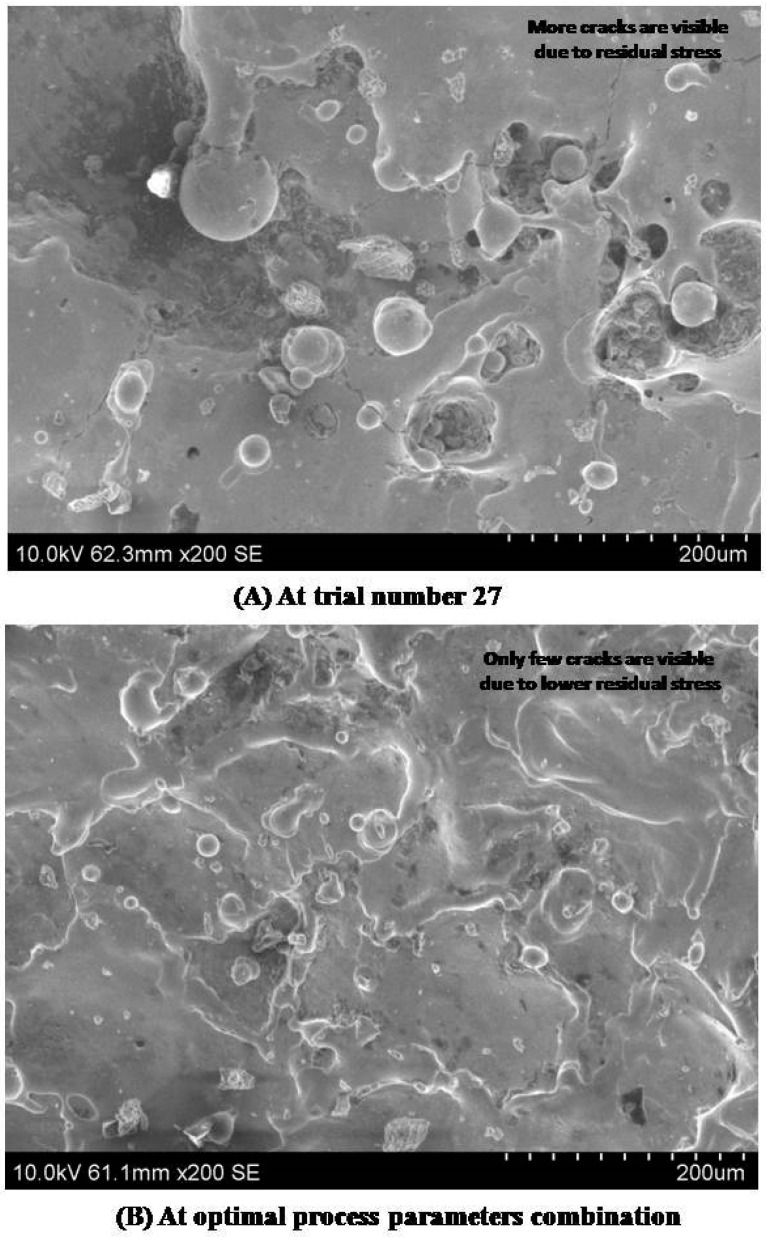
Comparison between surface morphology of machined specimens in WEDM process.

**Figure 7:**
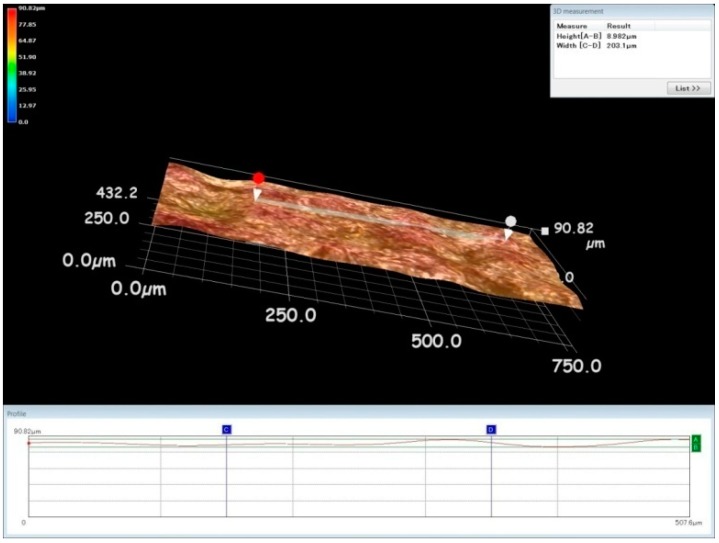
3D surface analysis of machined specimen in WEDM process.

Figure 6 shows the minimum white layer zone and a few cracks on the processed surface under the optimal process parameters combination. A higher white layer could increase the residual stress on the machined surface in the EDM process. The delivery of the higher pulse energy creates higher residual stress, which subsequently creates micro cracks. Due to this high residual stress, these cracks then propagate. This could affect the surface performance measures and fatigue life of titanium workpiece specimens in EDM. Dental implants should have lower residual stress to increase the life and quality of the products [1]. The surface quality of the specimens at optimal process parameters was observed to have fewer micro cracks, as shown in Figure 6. Hence, it was proved that the fatigue life of the machined components could be considerably enhanced by adopting the proposed MCDM technique. Dental implants should have considerably lower surface roughness. The surface roughness of the titanium alloy can be effectively modified by the pulse energy during the machining process. The heat affected zone (HAZ) can be viewed as the white region. It was noted that more HAZ was found in the trial with a high discharge energy combination than in the trial using the optimal combination. The surface roughness of machined specimens in the EDM process can be reduced by uniform and tiny craters on the processed surface [17]. In Figure 7, the distance between C and D indicates the evaluation length of the surface roughness measurement, while the distance between A and B specifies the maximum peak value of roughness. The average value of roughness was inferred from the figure itself. It was observed that the surface roughness could be effectively reduced by incorporating the optimal input parameters combination in the EDM process due to the optimal pulse energy as shown in Figure 7.

## 5. Conclusions

The TGRA method was used to assess the optimal factors combination in obtaining optimal surface measures such as wire wear ratio, MH and AWLT when machining titanium (α-β) alloy with the WEDM process. The following conclusions were made:

- In achieving better quality measures, the optimal electrical factors amongst the existing factor combinations were found to be gap voltage (70 V), discharge current (15 A) and duty factor (0.6).
- The maximum high-low grade value shows that the wire electrode affects the surface measures due to its significance in determining spark energy in WEDM.
- Using a TGRA based MCDM approach, the surface quality analysis has also shown that the optimal input factors combination significantly contributes to improving the quality of the machined surface.

## Figures and Tables

**Table 1:** Selection of Process factors.

Control Factor	Level I	Level II	Level III	Unit
T_on_	110	120	130	µs
T_off_	30	40	50	µs
SV	40	60	80	V
W_b_	5	7	9	Kg
WE	Brass Wire Electrode (BWE)	Zinc coated Brass Wire Electrode (ZWE)	Diffused Brass Wire Electrode (DWE)	-
Wire diameter	0.25			mm
Wire feed rate	4			m/min
Dielectric medium	Deionized water			-
Dielectric flow rate	1.2			bar
Peak current	16			A

**Table 2:** OA with performance measures.

Trial	T_on_	T_off_	SV	WT	WE	WWR	MH	AWLT
1	110	30	40	5	BWE	0.1666	516.76	5.1
2	110	30	40	5	ZWE	0.0909	465.2	2.11
3	110	30	40	5	DWE	0.0686	494.86	2.11
4	110	40	60	7	BWE	0.1248	518.3	2.72
5	110	40	60	7	ZWE	0.0454	500.33	1.85
6	110	40	60	7	DWE	0.1111	393.9	1.75
7	110	50	80	9	BWE	0.1682	514.2	5.33
8	110	50	80	9	ZWE	0.0919	445.4	3.43
9	110	50	80	9	DWE	0.0258	425.86	2.401
10	120	30	60	9	BWE	0.1686	429.9	3.17
11	120	30	60	9	ZWE	0.0908	466.1	0.55
12	120	30	60	9	DWE	0.0682	663.16	4.28
13	120	40	80	5	BWE	0.125	425.76	2.34
14	120	40	80	5	ZWE	0.0929	487.1	2.72
15	120	40	80	5	DWE	0.0222	421.73	4.59
16	120	50	40	7	BWE	0.125	460.26	5.67
17	120	50	40	7	ZWE	0.045	557.73	1.88
18	120	50	40	7	DWE	0.1121	543	5.84
19	130	30	80	7	BWE	0.125	401.96	3.73
20	130	30	80	7	ZWE	0.1165	563.26	2.55
21	130	30	80	7	DWE	0.1107	401.3	4.31
22	130	40	40	9	BWE	0.125	512.13	3.36
23	130	40	40	9	ZWE	0.0454	496.13	3.08
24	130	40	40	9	DWE	0.0666	508.96	3.54
25	130	50	60	5	BWE	0.125	366.46	2.77
26	130	50	60	5	ZWE	0.1365	534.8	2.37
27	130	50	60	5	DWE	0.1131	478.73	2.35
Mean						0.10025556	481.233	3.18152
Standard deviation						0.04096588	64.0385	1.3149
Standard error						0.00788412	12.3246	0.25306

**Table 3:** S/N value with its normalized value.

Trial No.	WWR		MH		AWLT	
	S/N Ratio	N S/N Ratio	S/N Ratio	N S/N Ratio	S/N Ratio	N S/N Ratio
1.	1.55665	0.994114	5.426578	0.579453	−1.41514	0.942649
2.	2.082872	0.695296	5.335279	0.402238	−0.64856	0.569094
3.	2.327352	0.556466	5.388965	0.506444	−0.64856	0.569094
4.	1.807571	0.851627	5.429162	0.58447	−0.86914	0.67658
5.	2.685888	0.352869	5.398513	0.524978	−0.53434	0.513433
6.	1.908572	0.794273	5.190772	0.121741	−0.48608	0.489912
7.	1.548348	0.998829	5.422264	0.57108	−1.45345	0.96132
8.	2.073369	0.700692	5.2975	0.328907	−1.07059	0.774748
9.	3.176761	0.074124	5.258534	0.25327	−0.76078	0.623779
10.	1.546285	1	5.266735	0.269189	−1.00212	0.741382
11.	2.083828	0.694753	5.336958	0.405496	0.519275	0
12.	2.332431	0.553582	5.643237	1	−1.26289	0.868456
13.	1.80618	0.852417	5.25833	0.252874	−0.73843	0.612886
14.	2.063969	0.70603	5.375236	0.479796	−0.86914	0.67658
15.	3.307294	0	5.250069	0.23684	−1.32363	0.898054
16.	1.80618	0.852417	5.326006	0.384238	−1.50717	0.987494
17.	2.693575	0.348504	5.492848	0.708087	−0.54832	0.520242
18.	1.900789	0.798693	5.4696	0.662961	−1.53283	1
19.	1.80618	0.852417	5.208366	0.155891	−1.14342	0.810238
20.	1.867348	0.817682	5.501418	0.724721	−0.81308	0.649263
21.	1.911705	0.792494	5.206938	0.153121	−1.26895	0.871412
22.	1.80618	0.852417	5.41876	0.564279	−1.05268	0.76602
23.	2.685888	0.352869	5.391191	0.510765	−0.9771	0.729191
24.	2.353052	0.541873	5.413367	0.55381	−1.09801	0.788109
25.	1.80618	0.852417	5.128053	0	−0.88496	0.68429
26.	1.729735	0.895827	5.456383	0.637306	−0.7495	0.618278
27.	1.893075	0.803073	5.360181	0.450574	−0.74214	0.614691

**Table 4:** GR coefficient with its rank.

No.	GR Coefficient			GR Grade
	WWR	MH	AWLT	
1.	0.988365	0.543155	0.897101	0.809541
2.	0.621346	0.455472	0.537111	0.537976
3.	0.529923	0.503243	0.537111	0.523426
4.	0.771161	0.546132	0.607223	0.641505
5.	0.43587	0.512809	0.506808	0.485162
6.	0.708489	0.362776	0.495006	0.522091
7.	0.997662	0.538259	0.928195	0.821372
8.	0.625541	0.426951	0.689415	0.580636
9.	0.350662	0.401049	0.570632	0.440781
10.	1	0.406236	0.659093	0.688443
11.	0.620927	0.456828	0.333333	0.470363
12.	0.528308	1	0.79171	0.773339
13.	0.772102	0.400922	0.563626	0.578883
14.	0.629747	0.490098	0.607223	0.575689
15.	0.333333	0.395833	0.830639	0.519935
16.	0.772102	0.448124	0.975598	0.731941
17.	0.434218	0.631382	0.51033	0.52531
18.	0.712954	0.597343	1	0.770099
19.	0.772102	0.371994	0.724887	0.622994
20.	0.732796	0.644929	0.587726	0.65515
21.	0.706708	0.371229	0.795434	0.624457
22.	0.772102	0.534347	0.681218	0.662556
23.	0.43587	0.505441	0.648669	0.529993
24.	0.521851	0.528435	0.702354	0.584214
25.	0.772102	0.333333	0.612963	0.572799
26.	0.827577	0.57958	0.567072	0.658076
27.	0.717436	0.476451	0.564775	0.58622

**Table 5:** Average GR grade for input factors.

Factor Notation	Average GR Grade			High-Low
	1	2	3	
**T_on_**	0.5958	0.6260	0.6107	0.0302
**T_off_**	0.5706	0.5667	0.6319	0.0652
**SV**	0.6306	0.5998	0.6022	0.0308
**WT**	0.5958	0.6199	0.6169	0.0240
**WE**	0.6811	0.5576	0.5938	0.1235

Total mean GR grade = 0.6066.
